# Temporary Resection of the Skull

**Published:** 1890-06-14

**Authors:** 


### SURGERY.

### TEMPORARY RESECTION OF THE SKULL.

So long ago as 1863 Dr. Julius Wolff, of Berlin, after some successful experimental operations on animals, proposed to substitute for trephining a procedure which would be free from the drawback attendant on the ordinary operation, the inconvenience, indeed the danger, of leaving a portion of the surface of the brain unprotected except by skin. Feasible and rational as it appears, the suggestion was not acted on by himself or others until last year, when Dr. W. Wagner, of Königshütten, in Upper Silesia, performed it with perfect success, and indeed with greater ease than he had anticipated. He first made an incision through the scalp and subcutaneous structures in shape like a capital omega, Ω, *i.e.*, an incomplete circle. Dissecting off and turning back the flap, he next carried an incision through the periosteum at about a quarter or a third of an inch from the margin of the skin. Then with a fine sharp chisel he divided the outer table keeping the point of the instrument directed obliquely inwards, so that the section of the inner table should be somewhat smaller than that of the outer. The bridge, or isthmus, of bone between the terminations of the Ω-shaped incision was then cautiously divided subcutaneously with the chisel, and the operation concluded by replacing the bone and carefully uniting the integuments with sutures. The position of the isthmus of undivided integuments, he observes, must be determined by the course and direction of the arteries of the scalp, that these may be left to maintain the nutrition of the part. The oblique division of the bone leaves a rim which prevents the sinking of the resected portion. He recommends the use of fine chisels in preference to circular saws or other instruments.

Dr. Müller, of Aachen, suggests that the outer table alone need be removed, the diploe and inner table being left, and the latter simply cracked or "nicked" in cases where the removal of pressure or the escape of fluid is called for, and not the evacuation of a deep abscess or excision of a tumour.

